# A pangenome approach-based loop-mediated isothermal amplification assay for the specific and early detection of *Bordetella pertussis*

**DOI:** 10.1038/s41598-023-29773-9

**Published:** 2023-03-16

**Authors:** Eduardo Juscamayta-López, Faviola Valdivia, María Pía Soto, Brenda Nureña, Helen Horna

**Affiliations:** 1 Centro Nacional de Salud Pública, Instituto Nacional de Salud, Lima, Perú; 2 Facultad de Salud Pública y Administración (GA, AGL), Universidad Peruana Cayetano Heredia, Lima, Perú

**Keywords:** Genetic techniques, Genetic testing, Infectious-disease diagnostics

## Abstract

Despite widespread vaccination, *Bordetella pertussis* continues to cause pertussis infections worldwide, leaving infants at the highest risk of severe illness and death, while people around them are likely the main sources of infection and rapidly spread the disease. Rapid and less complex molecular testing for the specific and timely diagnosis of pertussis remains a challenge that could help to prevent the disease from worsening and prevent its transmission. We aimed to develop and validate a colorimetric loop-mediated isothermal amplification (LAMP) assay using a new target uvrD_2 informed by the pangenome for the specific and early detection of *B. pertussis*. Compared to that of multitarget quantitative polymerase chain reaction (multitarget qPCR) using a large clinical DNA specimen (n = 600), the diagnostic sensitivity and specificity of the uvrD_2 LAMP assay were 100.0% and 98.6%, respectively, with a 99.7% degree of agreement between the two assays. The novel colorimetric uvrD_2 LAMP assay is highly sensitive and specific for detecting *B. pertussis* DNA in nasopharyngeal swabs and showed similar diagnostic accuracy to complex and high-cost multitarget qPCR, but it is faster, simpler, and inexpensive, which makes it very helpful for the reliable and timely diagnosis of pertussis in primary health care and resource-limited settings.

## Introduction

Pertussis is a highly contagious respiratory disease caused by *Bordetella pertussis* and a substantial cause of infant mortality and morbidity^1^. Pertussis accounts for approximately 24.1 million cases and 160,700 deaths among children under 5 years of age^2^, and its clinical manifestation is more severe in infants younger than one year of age, with high rates of hospitalization and risk of death^3,4^. Thus, it has become a major public health concern. Accurate and timely diagnosis of pertussis infections is critical to prevent the disease from worsening and to interrupt the transmission of the bacteria in the population. However, pertussis diagnosis is a major challenge, as there is variability in the clinical case definition, which makes earlier detection difficult^5^. Additionally, other respiratory pathogens cause similar symptoms, and the clinical presentation of the disease can vary with age and vaccination status^6^. Therefore, laboratory tests are required to confirm suspicious clinical cases, and culture is considered the standard reference method for the diagnosis of pertussis due to its 100% specificity. Nevertheless, this method has a very low sensitivity (12–60%), and results are available in 1 to 2 weeks^7^. In addition to requiring highly qualified personnel, different factors affect the viability and isolation of the pathogen, such as the swab material, collection time and transport medium of the nasopharyngeal sample, the enrichment medium and whether the patient has been recently vaccinated or received antibiotics against pertussis^8,9^, all of which make a complex and variable test. Serological methods based on pertussis toxin (PT) IgG enzyme-linked immunosorbent assay (ELISA) have been analytically validated and used to confirm pertussis infections during outbreaks^10,11^. In a validation study using culture as the gold standard, anti-PT IgG ELISA was found to be more sensitive in sera collected from patients more than two weeks from the onset of cough than during the first two weeks from the onset of cough (60%, 95% CI 17.1–100% vs. 16.7%, 95% CI 0.0–37.8%)^12^; therefore, this assay is not adequate for the early diagnosis of illness in a susceptible population. Furthermore, other factors limit the performance of the method, including the use of paired sera in the acute and convalescent phases, recent vaccination status, differences in cutoff values and the occurrence of cross-reactions^13^.

Molecular methods based on quantitative polymerase chain reaction (qPCR) have been the most used for the rapid and sensitive diagnosis of pertussis^14^. Unlike culture, qPCR assay results can be obtained in a short time (2–24 h). Furthermore, it does not require that the pathogen be viable to be positive, presenting a sensitivity and specificity between 70–99% and 86–100%, respectively, when performed less than four weeks from the onset of symptoms^7,15^. Multiplex qPCR assays are being used for the detection and differentiation of *Bordetella* species, targeting insertion sequences (ISs) because of their high genomic copy number. However, studies have reported that IS*481* is present not only in *B. pertussis* but also in *Bordetella holmesii*^16^, while IS*1001* is included in the genome of *Bordetella parapertussis*^17^, and IS*1002* is present in *B. pertussis* and *B. parapertussis*. In addition, these markers have also been detected in some strains of *Bordetella bronchiseptica*^18^ and hence are not species specific. In contrast, single-copy targets, such as the pertussis toxin S1 subunit (*ptx*S1) promoter region (*ptx*S1-Pr), are specific for *B. pertussis* but have lower sensitivity than insertion sequences^19,20^. The Centers for Disease Control and Prevention (CDC, Atlanta, USA) have developed a multitarget quantitative PCR (multitarget qPCR) assay that combines a simple assay targeting *ptx*S1 and a multiplex assay based on IS*481*, pIS*1001*, and hIS*1001* for the detection and differentiation of *B. pertussis, B. parapertussis*, and *B. holmesii*. The multitarget qPCR assay has been evaluated using 197 clinical samples obtained during an outbreak, yielding efficiencies that ranged from 81 to 99% for the multiplex assay and > 99% for the singleplex assay, with an increase in the specificity in the diagnosis of *B. pertussis* infections compared to other IS-based multiplex assays^21,22^. Although multitarget qPCR has good performance and provides relatively fast results, the combined use of multiple targets increases the risk of misinterpretation, as shown by a study where multiplex PCR based on IS*481* and IS*1002*, using a sample of *B. pertussis* DNA at a low concentration, resulted in an amplification signal only for IS*481*, which was interpreted as positive for *B. holmesii* DNA when *B. pertussis* was the causative agent^23^. Another study reported differences in the agreement between laboratory cycle threshold values (concordance correlation coefficients = 0.65–0.84) and changes in interpretations of results when using multiplex qPCR based on these targets^24^. Moreover, this method is laborious and requires sophisticated equipment, specialized personnel, and expensive consumables, which are hardly available in resource-limited settings. This makes the timely management of patients and the prevention of further transmission difficult.

An alternative method is loop-mediated isothermal amplification (LAMP), which has been demonstrated to be fast, simple and sensitive in the diagnosis of respiratory infectious diseases, including tuberculosis^25^, influenza^26^ and COVID-19^27^. LAMP amplifies DNA under isothermal conditions using only a set of six primers and DNA polymerase with displacement activity^28^. LAMP assays based on *ptx*S1-Pr and IS*481* have been proposed for the diagnostic detection of *B. pertussis*. Nevertheless, these targets are the same as those being used for qPCR and still have problems of sensitivity and cross-reactivity with other *Bordetella* species^29^. This increases the rate of false negatives and positives, leading to unreliable diagnostic results. In addition, the performance of these assays is still subject to discussion owing to the low numbers of samples tested thus far^30^. There is an ongoing need to develop a new LAMP assay based on a more specific and sensitive target to provide an accurate diagnosis and timely treatment of patients infected by *B. pertussis*.

Here, we developed and validated a molecular assay based on one-step colorimetric LAMP using a new target screened from the classical *Bordetella* pangenome and lyophilized LAMP reagents for the specific and sensitive detection of *B. pertussis* DNA isolated from nasopharyngeal swabs collected from confirmed pertussis patients at the National Institute of Health-Peru (NIH-Peru). First, we sequenced the genomes of *B. pertussis* circulating in Peru. Using these sequences together with other classical *Bordetella* subspecies genomic sequences in publicly available databases, we identified potential *B. pertussis*-specific targets by pangenome analysis. Then, we used 600 clinical DNA samples from individuals tested for pertussis to validate our colorimetric LAMP assay compared to multitarget qPCR (gold standard). In this regard, we present a new portable and colorimetric LAMP assay informed by the pangenome with high performance, which allows the accurate and timely naked eye detection of *Bordetella pertussis* in a low-resource setting.

## Methods

### Study design and setting

This was a retrospective study to develop and validate a colorimetric LAMP assay based on a new target identified by pangenome analysis for the specific and early detection of *B. pertussis* DNA in clinical DNA samples. To validate the assay, we used DNA samples isolated from nasopharyngeal specimens collected from patients who had been tested for pertussis at NIH-Peru from January 2018 to December 2019. This study was conducted at NIH-Peru.

### Sample collection and DNA extraction

We obtained DNA from nasopharyngeal swabs from patients with suspected pertussis who were referred to NIH-Peru for diagnostic confirmation by multitarget qPCR (gold standard). Bacterial DNA from 200 μl of clinical sample had previously been extracted using the PureLink^®^ Genomic DNA Mini Kit (Invitrogen, Waltham, Massachusetts, USA) following the manufacturer’s protocols, eluted in 60 μl of elution buffer, and examined by multitarget qPCR. The remaining DNA samples were stored at − 80 °C. We randomly selected DNA from clinical samples that were positive (n = 300) and negative (n = 300) for *B. pertussis* DNA according to multitarget qPCR (gold standard) from January 2018 to December 2019. The selected DNA samples were stored at − 80 °C until further processing.

### Ethics statement

This study was reviewed and approved by the Ethics in Research Committee of the National Institute of Health of Peru (reference numbers OI-032-18; OT-024-19) and Universidad Peruana Cayetano Heredia (Reference Number 103935). Written informed consent for participation was not required due to the retrospective nature of this study in accordance with national legislation and institutional requirements. The informed consent was waived by the Ethics in Research Committee of the National Institute of Health of Peru due to retrospective nature of study.

### *Bordetella pertussis* strains and whole-genome sequencing (WGS)

Fourteen *B. pertussis* strains sequenced in this study are listed in online Supplementary Table S1. These strains had previously been isolated from nasopharyngeal swabs collected from pertussis-suspected individuals reported in 2012 in Perú^31^. All 14 *B. pertussis* strains were retrieved from storage at − 80 °C, inoculated onto Jones-Kendrick charcoal agar plates, and incubated at 37 °C for 72 h. Briefly, colonies were harvested from each plate and resuspended in 500 µl of phosphate-buffered saline (PBS). The cell suspension was adjusted to an OD 600 nm = ⁓1.0 and pelleted in a microcentrifuge for 10 min at 5000 × *g*. Genomic DNA was extracted from pelleted cultures with the PureLink® Genomic DNA Mini Kit (Invitrogen, Waltham, Massachusetts, USA), and *B. pertussis* isolates were confirmed by multitarget qPCR.

WGS of *B. pertussis* isolates was performed using an Illumina MiSeq System at NIH-Peru. Libraries for paired-end sequencing were prepared with the Nextera XT DNA Library Preparation Kit (Illumina, San Diego, California, USA).

### Sequence quality assessment and genome assembly

The quality of the obtained sequences was evaluated using FastQC v0.11.9 (https://www.bioinformatics.babraham.ac.uk/projects/fastqc). Reads of low-quality, duplicated and adapter sequences were trimmed off with Trimmomatic v0.32^32^ using a Phred quality score cutoff of 20. Filtered reads were assembled de novo using SPAdes v.3.15.3^33^.

### Pangenome analysis and identification of *B. pertussis*-specific targets

All publicly available classical *Bordetella* subspecies genomes were downloaded from the PATRIC database (https://www.patricbrc.org/) as of January 5, 2021. Searches included *B. pertussis* (n = 847), *B. parapertussis* (n = 24) and *B. bronchiseptica* (n = 92) genomes. These genome sequences are listed in online Supplementary Table S1.

The 977 classical *Bordetella* genomes, including those from Peru, were annotated using Prokka v1.14.5^34^, and pangenome analysis was carried out with Roary v3.11.2^35^. The matrix with the presence and absence of core and accessory genes (pangenome) was used to identify *B. pertussis*-specific genes to be used as potential diagnostic targets. Using different libraries of R v4.0.5, we first cleaned the dataset by removing description variables and converted the pangenome to a binary presence/absence matrix using the dplyr v1.0.8 package (https://CRAN.R-project.org/package=dplyr). Then, we filtered genes that are present in *B. parapertussis* genomes from the binary matrix and created a clustered heatmap using the pheatmap v1.0.12 (https://CRAN.R-project.org/package=pheatmap) and dendextend v1.16.0 (https://CRAN.R-project.org/package=dendextend) packages. We performed hierarchical clustering to obtain gene clusters that inform us of what genes are present in most *B. pertussis* genomes but not in *B. bronchiseptica* genomes. Sequences of selected diagnostic targets were extracted with a bash script.

### LAMP primer design

We designed LAMP primers for each selected *B. pertussis*-specific gene using Primer Explorer v5 (https://primerexplorer.jp/e/). Sets of LAMP primers with the best parameters were selected and synthesized by Bio Basic (Bio Basic, Markham, Ontario, Canada). Each set included two outer primers (F3 and B3), two inner primers (forward inner primer [FIP] and backward inner primer [BIP]), and loop forward (LF) and loop backward (LB) primers (online Supplementary Table S2).

### One-step colorimetric LAMP assay

The LAMP assays were performed in a Loopamp real-time turbidimeter (LA-500; Eiken Chemical Co., Ltd., Tokyo, Japan) using lyophilized LAMP reagents from the Loopamp RNA/DNA amplification reagent D kit (Eiken Chemical Co., Ltd., Tokyo, Japan). The LAMP reaction mixture (25 µL) contained 18 µL of rehydrated reagents, 1.6 µmol L^−1^ each of the inner primers FIP and BIP, 0.2 µmol L^−1^ each of the outer primers F3 and B3, 0.8 µmol L^−1^ each of the loop primers LF and LB, and 5 µL of template DNA. First, we individually evaluated each set of LAMP primers by incubating the reaction mixtures at 60 °C, 63 °C, 65 °C, and 67 °C for 60 min and then at 80 °C for 5 min to complete the reaction.

Second, we set up one-step colorimetric LAMP assays targeting the final target selected using the same conditions described above except that 5 µL of template DNA previously denatured at 96 °C for 5 min was used in the reaction. Optimization of LAMP was carried out in real time by determining the amplification time (in minutes) and turbidity at 650 nm using the LA-500 turbidimeter. A LAMP-positive reaction was considered when the turbidity reached 0.05 within 60 min and by the color change from brown to green and the typical ladder-like pattern of bands revealed by gel electrophoresis. To confirm the performance of the reagents and the absence of contamination, a *B. pertussis*-positive control (BPC) and a nontemplate control (NTC) were included in each assay.

### Multitarget qPCR assay

We used the multitarget qPCR assay previously described by Tatti et al.^21^ with slight modifications. In brief, the multiplex assay targeting the insertion sequences IS*481*, pIS*1001*, and hIS*1001* and human *rnaseP* (internal control) was performed in a Rotor-Gene Q (Qiagen, Germantown, Maryland, USA) using a 25-μl reaction mixture containing 5 μl of template DNA and the primers/probes in 1 × KAPA PROBE FAST qPCR Master Mix (Kapa Biosystems, Cape Town, South Africa). The PCR cycling parameters were as follows: 50 °C for 2 min and 95 °C for 10 min, followed by 45 cycles at 95 °C for 15 s and 60 °C for 1 min. The singleplex assay was based on pertussis toxin S1 subunit (*ptx*S1) and was performed according to amplification conditions described for the multiplex assay with the exception of an annealing temperature of 57 °C^15^. Interpretation of multitarget qPCR results was carried out using the diagnostic algorithm in online Supplementary Table S3. Primer and probe sequences, as well as optimized concentrations, are listed in online Supplementary Table S4.

### Analytical sensitivity of the colorimetric LAMP assay

The sensitivity of the optimized LAMP assay for detecting *B. pertussis* was determined using genomic DNA extracted from *B. pertussis* strain Tohama 1 (ATCC BAA-589), and the DNA concentration was calculated using a Qubit 4 Fluorometer (Thermo Fisher Scientific). DNA was adjusted to 20 ng/μl and converted to 10^6^ copies of genome equivalent per microliter (gEq × μl^−1^) using a standard of 4 Mb per *B. pertussis* genome. To determine the sensitivity of the LAMP assay in conditions that simulated the laboratory-based detection process, the 10^6^ copies gEq × μl^−1^ were serially diluted tenfold up to 1 copy gEq × μl^−1^ using a DNA pool obtained from nasopharyngeal swabs that were negative for *B. pertussis, B. parapertussis,* and *B. holmesii* DNA according to multitarget qPCR. The diluted DNA samples were previously denatured at 96 °C for 5 min and immediately tested in parallel by colorimetric LAMP and multitarget qPCR assays. The limit of detection (LoD) was determined by identifying the lowest concentration of *B. pertussis* DNA at which ≥ 95% of 10 replicates showed positive results.

Five microliters of each LAMP reaction was electrophoresed on a 2% agarose gel in 1 × TAE buffer (40 mM Tris, 20 mM acetic acid, 1 mM EDTA) at 110 V for 60 min. Reactions were considered positive for LAMP products if they had both a color change from brown to green and a ladder-like banding pattern on agarose gel after electrophoresis.

### Analytical specificity of the colorimetric LAMP assay

We evaluated the cross-specificity of the colorimetric LAMP assay using bacterial pathogens associated with respiratory infections, including *B. parapertussis* (Bpp)*, B. holmesii* (Bh)*, B. bronchiseptica* (Bb)*, Neisseria meningitidis* (Nm)*, Streptococcus pneumoniae* (Spn)*, Haemophilus influenzae* (Hinf)*, Klebsiella pneumoniae* (Kpn)*, Corynebacterium diphtheriae* (Cd), *Staphylococcus aureus* (Sa)*, Haemophilus parainfluenzae* (Hpi), *Pseudomonas aeruginosa* (Pae)*,* and *Escherichia coli* (Ec). We also included a set of respiratory viruses comprising influenza A (H1N1/H3N2) virus (FluA), influenza B (Victoria/Yamagata) virus (FluB), SARS-CoV-2 virus, human metapneumovirus (hMPV), respiratory syncytial A (RSV A) and B (RSV B) virus, and rhinovirus (RhV). Prior to this, DNA or RNA was quantified using a Qubit 4 Fluorometer (Thermo Fisher Scientific), and concentrations were adjusted to 1–2 ng/µL.

We also evaluated the analytical in silico specificity of the final primer set by means of BLASTn analysis using a local pathogen database that included closely related Bordetella species and other respiratory infection-associated viral and bacterial genomes (online Supplementary Table S5), according to the method described by Juscamayta-López et al.^27^.

### Evaluation of repeatability, reproducibility, and robustness

Precision was assessed using *B. pertussis* (ATCC BAA-589) DNA with concentrations ranging from 10^6^ copies gEq × μl^−1^ up to 10^3^ copies gEq × μl^−1^. Intra-assay (repeatability) and interassay (reproducibility) precision were calculated by means of the coefficient of variation (CV) and testing 3 and 6 replicates within and between runs, respectively, and by different operators^27^.

The robustness of the method was assessed by introducing small variations in concentrations of LAMP primers (0.75 × , 0.5 × , and 0.4 × regarding the optimal concentration) and testing at two different amplification temperatures (65 °C and 67 °C). Assays were performed using 10^5^ copies gEq × μl^−1^ in triplicate under the same operator, equipment, and laboratory^27^.

### Diagnostic validation using clinical samples

We evaluated the colorimetric LAMP assay based on the final primer set using 600 DNA samples obtained from nasopharyngeal swabs collected from individuals who tested positive (n = 300) and negative (n = 300) for *B. pertussis* DNA based on multitarget qPCR (gold standard). These samples were randomly selected and tested in parallel by both multitarget qPCR and LAMP assays.

The LAMP assays were carried out using 5 μL of DNA, previously denatured at 96 °C for 5 min, from each sample per reaction in the LA-500 turbidimeter, as described above. Clinical evaluation was performed in a blinded manner regarding the predefined pertussis status and the reference test results. LAMP was confirmed by visual judgment based on a color change from brown to green and when the turbidity reached 0.05 within 60 min. Sensitivity, specificity, positive predictive value (PPV) and negative predictive value (NPV)^36^ were calculated to assess the diagnostic accuracy of the colorimetric LAMP compared to multitarget qPCR (gold standard).

### Statistical analysis

Statistical analysis was performed using Stata/MP v15.0 and R v4.0.5. One-way analysis of variance (ANOVA) and Student’s t-test with significance p < 0.05 was used to determine statistical significance between the different times to positivity (Tp) of the LAMP primer sets, and between azithromycin ≤ 3 doses and 4–5 doses samples for cycle threshold (CT) values of *ptx*S1 multitarget qPCR, respectively. Precision was determined by obtaining mean time to positive values and standard deviations (SDs) of each set of replicates at a given concentration and calculating coefficients of variation (CV = SD/mean)^27^. Sensitivity, specificity, positive predictive value (PPV) and negative predictive value (NPV) were calculated by comparison between the results of the LAMP and multitarget qPCR (gold standard) assays using a 2 × 2 contingency table and 95% confidence interval (CI). The degree of agreement between the assays was estimated using kappa concordance coefficients (Cohen’s kappa; ≥ 0.75 was considered good) and percentage agreement (≥ 0.9 was considered good)^27,37^. The Pearson correlation coefficient was used to assess the strength of the relationship between the Tp and cycle threshold (CT) values of LAMP and multitarget qPCR for the targets IS*481* and *ptx*S1, respectively*.*

## Results

### Identification of *B. pertussis*-specific diagnostic targets informed by the pangenome

**Figure 1:**
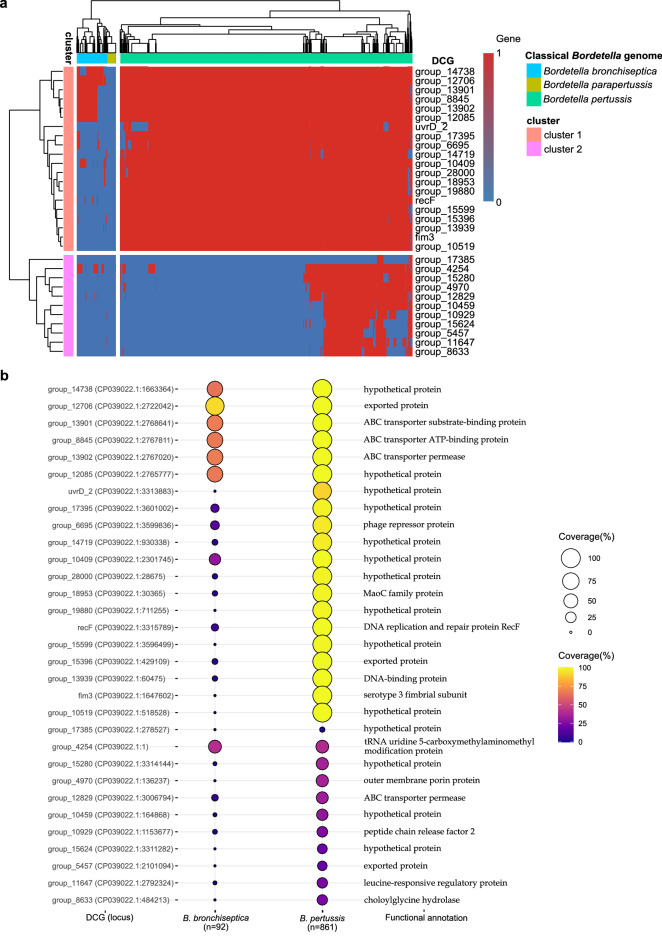
Screening of new pangenome-based diagnostic targets for specific detection of *B. pertussis*. (**a**) Heatmap and hierarchical clustering analysis of candidate diagnostic genes informed by the classical Bordetella pangenome (n = 977). (**b**) Functional annotation of candidate diagnostic genes and their coverage (presence or absence) in the *Bordetella bronchiseptica* and *Bordetella pertussis* genomes. DCG, diagnostic candidate gene; 1, presence; 0, absence. Figure 1a was created using pheatmap v1.0.12 and dendextend v1.16.0 packages for R v4.0.5. Figure 1b was generated using ggplot2 v3.3.6 package for R v4.0.5.

We analyzed the classical *Bordetella* pangenome (n = 977), including worldwide genomes of *B. parapertussis, B. bronchiseptica* and *B. pertussis*, and recently sequenced *B. pertussis* genomes isolated from Peru to select new diagnostic targets that were specific for *B. pertussis*. The pangenome of these species shared 1616 genes that included between 95 and 99% of genomes. Of these core genes, we managed to identify a total of 31 candidate genes for diagnosis that were present only in genomes from *B. pertussis* and some *B. bronchiseptica* genomes but not in *B. parapertussis* (Fig. 1a). Most diagnostic candidate genes encode hypothetical proteins (14/31), followed by outer membrane proteins associated with transporters (9/31) and proteins involved in metabolic and cellular processes (8/31) (Fig. 1b). Hierarchical clustering analysis showed two well differentiated clusters of these genes (Fig. 1a) that are present in 90–100% of *B. pertussis* genomes (n = 20, Cluster 1) and only in 3–40% of genomes of this pathogen (n = 11, Cluster 2). Both clusters included genes that are absent (0%) and present (1–85%) in *B. bronchiseptica* genomes (Fig. 1b).

### LAMP primer design and evaluation

**Figure 2:**
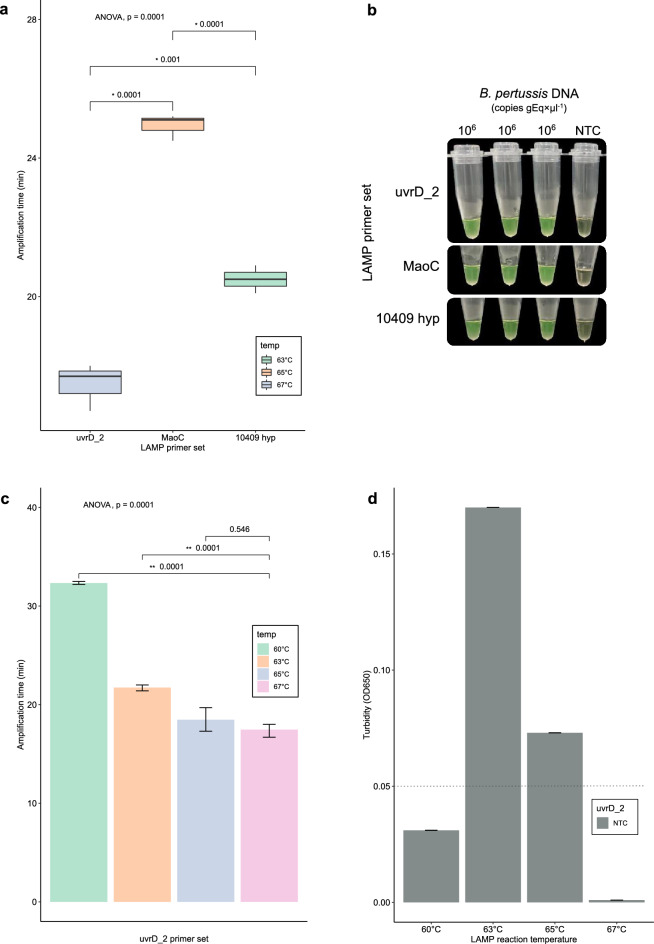
Effect of temperature and uvrD_2, MaoC, and 10,409 hyp primer sets on colorimetric LAMP assay performance. (**a**) Box plot depicting the amplification time (time to positivity) at the optimized temperature by the LAMP primer set. Boxes indicate the first to third quartile (interquartile range, IQR) with median (central line), and whiskers encompass 95% of the data. *P ≤ 0.001 shown at the top of the boxes. Significance was analyzed by one-way analysis of variance (ANOVA) of the mean amplification time from independent experiments performed in triplicate. (**b**) Colorimetric LAMP assays using uvrD_2, MaoC, and 10,409 hyp primer sets, in triplicate, at the optimized temperature for the detection of *Bordetella pertussis* DNA. (**c**) Evaluation of the uvrD_2 primer set at different temperatures. Error bars represent the standard deviations of the mean amplification time from independent experiments performed in triplicate. **P < 0.001. Significance was analyzed by one-way analysis of variance (ANOVA). (**d**) Turbidity of the NTC LAMP reaction analyzed in (**c**). The dotted line shows the threshold turbidity at 650 nm. A LAMP-positive reaction was considered when the turbidity increased above 0.05 within 60 min and the color changed from brown to green. LAMP assays were performed with *B. pertussis* DNA (positive control) obtained from *B. pertussis* strain Tohama 1 (ATCC BAA-589) at 10^6^ copies of genome equivalent per microliter (gEq × μl^−1^). *NTC* negative nontemplate control.

The higher the diagnostic candidate gene coverage in *B. pertussis* strains, the more trustworthy the designed primers for detecting any isolates of *B. pertussis*. Thus, LAMP primers were designed from potential diagnostic targets from Cluster 1, resulting in 3 optimized LAMP primer sets that targeted the *uvrD_2* (uvrD_2), *group_18953* (MaoC), and *group_10409* (10409_hyp) genes that encode for hypothetical proteins (uvrD_2 and 10409_hyp) and MaoC family protein (MaoC). These targets provide nearly 100% *B. pertussis* coverage (n = 861). The *group_18953* and *group_10409* genes covered 3 and 30%, respectively, of the 92 *B. bronchiseptica* genomes, while *uvrD_2* did not match any *B. bronchiseptica* strains analyzed (Fig. 1b). Each set of LAMP primers was individually evaluated at different temperatures in LAMP assays based on the lowest positive amplification time (time to positivity) for the detection of *B. pertussis* DNA. The best detection time was achieved using the uvrD_2 (μ = 17.47 ± 0.39 min) primer set at an optimal temperature of 67 °C, whereas using the MaoC and 10409_hyp primer sets obtained a time to positive average of 24.93 ± 0.22 min and 20.50 ± 0.23 min at an optimal temperature of 65 and 63 °C, respectively (Fig. 2a and b). uvrD_2 was the unique LAMP primer set able to detect *B. pertussis* DNA at different temperatures that ranged from 60 to 67 °C, suggesting that it is more robust than the other primers (Fig. 2c). However, the LAMP reaction of the NTC using uvrD_2 showed no turbidity at a temperature of 67 °C (threshold turbidity = 0.05 at 650 nm), while at other temperatures, the LAMP reaction showed minimal or high turbidity above the threshold (Fig. 2d).

**Figure 3:**
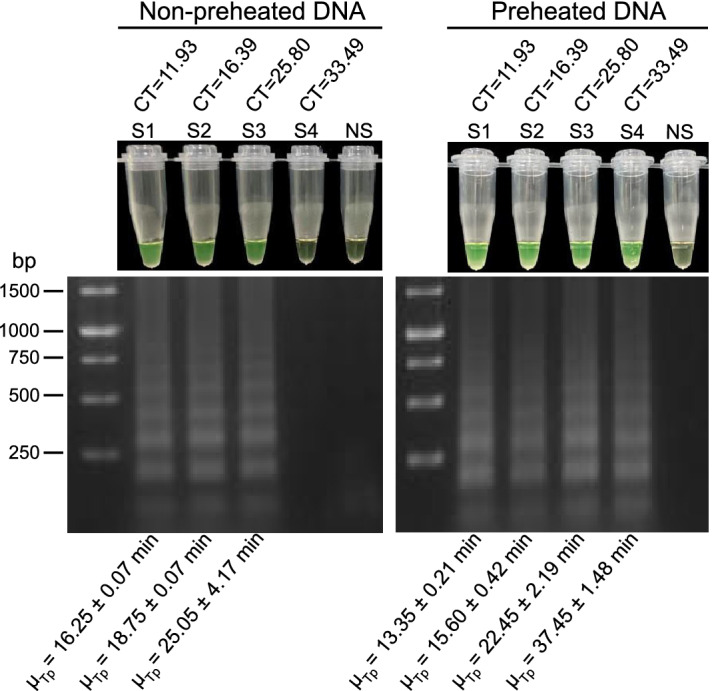
Evaluation of the uvrD_2-based LAMP assay using preheated and nonpreheated template DNA from a clinical sample panel with a bacterial load gradient. Preheated DNA consisted of denaturing template DNA at 96 °C for 5 min prior to loading of the sample for the LAMP reaction. Reactions were considered positive for LAMP products if they had both a color change from brown to green and a ladder-like banding pattern on agarose gel after electrophoresis. S1–S4, clinical samples positive for *B. pertussis* DNA with different cycle threshold (CT) values obtained by multitarget qPCR; NS, clinical samples negative for *B. pertussis* DNA; bp, base pair; µ_Tp_, mean time to positivity (Tp) in minutes (min) in the LAMP reaction (mean ± SD). Original gels are presented in online Supplementary Fig. S2.

Since it has been shown that the LAMP assay using denatured template DNA is more sensitive than using a nondenatured template^38^, we evaluated the uvrD_2-based LAMP assay at the optimized temperature using a panel of *B. pertussis* DNA without preheating and previously denatured at 96 °C for 5 min obtained from nasopharyngeal swabs (n = 4) with a bacterial load gradient. To evaluate whether the denatured genetic background of a clinical sample could cross-react with the final LAMP primer set, we also included clinical samples that were negative for *B. pertussis* DNA by multitarget qPCR as a negative control. The LAMP assay either with or without denatured DNA was able to detect *B. pertussis* DNA in clinical samples (CT = 16.39 − 25.8), while LAMP with preheated DNA managed to amplify *B. pertussis* DNA in a sample with a low bacterial load (CT = 33.49), as evidenced by the brown to green color change and the ladder-like banding pattern revealed by gel electrophoresis analysis (Fig. 3). Hence, previously denatured DNA was used for all LAMP assays. Negative controls did not react in the LAMP reaction, as evidenced by the lack of color change of the solution and absence of the ladder-like banding pattern (Fig. 3).

### Limit of detection and specificity of the LAMP assay

**Figure 4:**
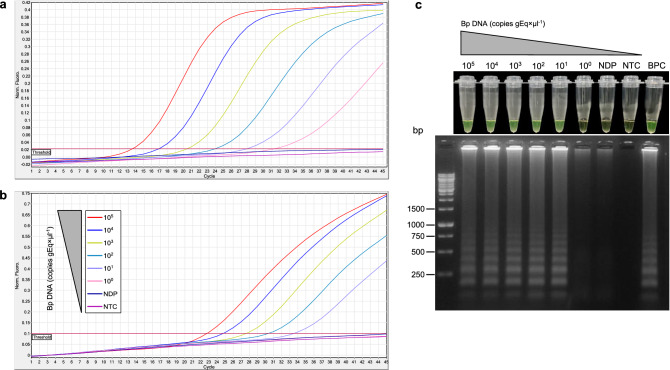
Analytical sensitivity of the colorimetric uvrD_2 LAMP assay using nasopharyngeal swab specimens spiked with *Bordetella pertussis* DNA. The limit of detection (LoD) was assessed using *B. pertussis* DNA serially diluted tenfold from 10^5^ to 1 copy of genome equivalent per microliter (gEq × μl-1) using nasopharyngeal swabs that were negative for *B. pertussis* (Bp), *B. parapertussis*, and *B. holmesii* DNA. All log-dilutions were analyzed in parallel by multitarget qPCR based on (**a**) IS*481* and (**b**) *ptx*S1 and (**c**) colorimetric LAMP assays. The LoD of the colorimetric uvrD_2 LAMP assay was 10 copies gEq × μl-1, which was defined as the lowest concentration of *B. pertussis* DNA at which ≥ 95% of replicates (n = 10) showed positive results. LAMP reactions were considered positive for *B. pertussis* DNA if they had both a color change from brown to green and a ladder-like banding pattern on agarose gel after electrophoresis. NDP, DNA pool obtained from nasopharyngeal swabs that were negative for *B. pertussis*, *B. parapertussis*, and *B. holmesii* DNA; *NTC* negative nontemplate control, *BPC* *B. pertussis* positive control, *bp* base pair.

The LoD of colorimetric LAMP was tested using tenfold serially diluted *B. pertussis* genomic DNA from 10^5^ to 1 copy gEq × μl^−1^ and compared with results from multitarget qPCR. As shown in Fig. 4c, LAMP was positive from 10^5^ to 10 copies gEq × μl^−1^ in < 30 min, as was visually judged by a brown to green color change, and the amplified products showed a ladder-like banding pattern, as revealed by agarose gel electrophoresis. Therefore, the 95% LoD of the uvrD_2 LAMP assay was 10 copies gEq × μl^−1^, which was the lowest concentration of *B. pertussis* DNA at which ≥ 95% of replicates showed positive results. In contrast, the LoDs for IS*481* with qPCR and *ptx*S1 with qPCR were 1 and 10 copies gEq × μl^−1^, respectively (Fig. 4a and b).

**Figure 5:**
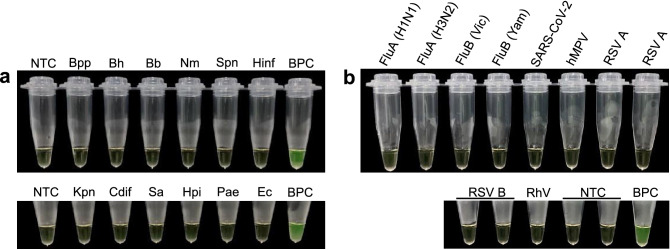
Analytical specificity of the colorimetric LAMP assay using (**a**) bacterial and (**b**) viral pathogens associated with respiratory infections. *Bpp* *Bordetella parapertussis*, *Bh* *Bordetella holmesii*, *Bb* *Bordetella bronchiseptica*, *Nm* *Neisseria meningitides*, *Spn* *Streptococcus pneumoniae*, *Hinf* *Haemophilus influenza*, *Kpn* *Klebsiella pneumoniae*, *Cdif* *Corynebacterium diphtheria*, *Sa* *Staphylococcus aureus*, *Hpi* *Haemophilus parainfluenzae*, *Pae* *Pseudomonas aeruginosa*; *Ec* *Escherichia coli*, *Flu* influenza virus, *Vict* Victoria, *Yam* Yamagata, *SARS-CoV-2* severe acute respiratory syndrome coronavirus 2, *hMPV* human metapneumovirus, *RSV* respiratory syncytial virus, *RhV* rhinovirus, *NTC* negative nontemplate control, *BPC* *B. pertussis* positive control.

In addition, no cross-reactivity was detected with other respiratory bacteria, namely, Bpp*,* Bh*,* Bb*,* Nm*,* Spn*,* Hinf*,* Kpn*,* Cd, Sa*,* Hpi, Pae*,* or Ec (Fig. 5a). Similarly, no amplification was observed with other respiratory viruses, including FluA (H1N1/H3N2), FluB (Victoria/Yamagata), SARS-CoV-2, hMPV, RSV A, RSV B, and RhV (Fig. 5b). These results were confirmed by in silico cross-reactivity analysis that revealed no match of any LAMP primer sequences with any genomes of respiratory pathogens likely circulating in Peru (online Supplementary Table S5).

### Repeatability, reproducibility and robustness

The average CV values of the colorimetric LAMP assay time to positivity regarding intra- and interassay precision were 5.09% and 4.14%, respectively (Tables 1 and 2). Intra-assay precision for the four *B. pertussis* DNA concentrations ranged from 1.83% at 10^4^ to 7.29% at 10^3^ copies gEq × μl^−1^. The assay proved to have high precision between runs at high and medium concentrations, while that at low concentrations showed a relatively higher value, with interassay precision ranging from 0.17% at 10^6^ to 11.73% at 10^3^ copies gEq × μl^−1^.

**Table 1:** Intra-assay precision of the colorimetric LAMP assay.

*B. pertussis* DNA copies gEq × μl^1^	No. of replicates	Mean Tp (min)	SD	CV (%)
10^6^	3	16.23	0.80	4.94
10^5^	3	19.30	1.22	6.30
10^4^	3	22.13	0.40	1.83
10^3^	3	30.23	2.20	7.29
			Mean CV (%)	5.09

*Tp* time to positivity, *SD* standard deviation, *CV* coefficient of variation, *gEq* genome equivalent.

**Table 2:** Interassay precision of the colorimetric LAMP assay.

*B. pertussis* DNA copies gEq × μl^1^	No. of replicates	Mean Tp (min)	SD	CV (%)
10^6^	6	16.25	0.03	0.17
10^5^	6	19.27	0.04	0.18
10^4^	6	21.45	0.96	4.48
10^3^	6	27.91	3.27	11.73
			Mean CV (%)	4.14

*Tp* time to positivity, *SD* standard deviation, *CV* coefficient of variation, *gEq* genome equivalent.

The colorimetric LAMP assay was highly robust to variations in the primer concentrations regarding the optimal concentration, amplifying *B. pertussis* DNA at 0.75 × , 0.5 ×  and 0.4 × at 65 °C and 67 °C, as evidenced by the color change from brown to green.

### Colorimetric LAMP diagnostic performance

**Figure 6:**
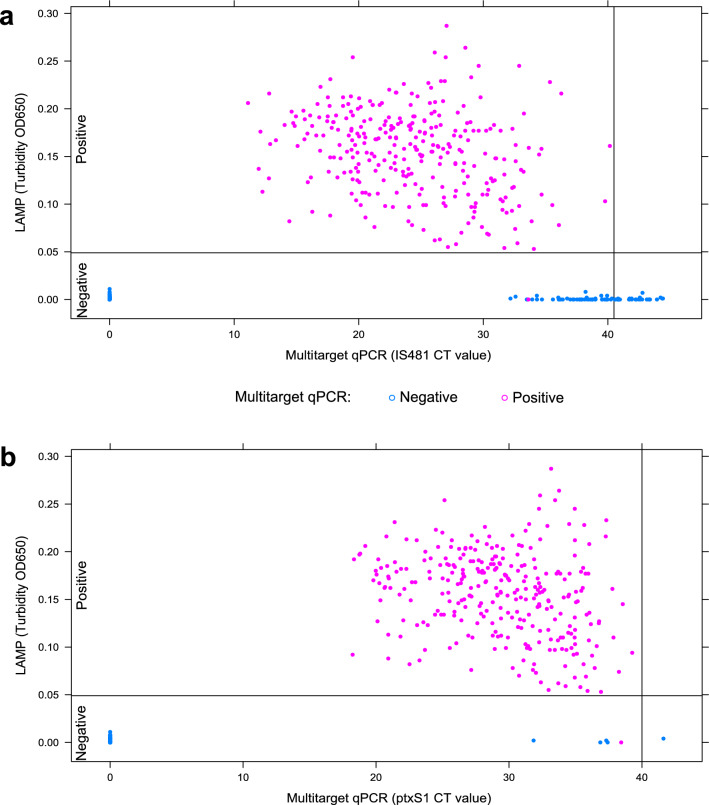
Detection based on colorimetric LAMP of *Bordetella pertussis* DNA compared to multitarget qPCR using clinical samples. Scatter plots show a comparison between the testing results of LAMP and multitarget qPCR assays for DNA samples obtained from nasopharyngeal swabs (n = 600). The turbidity of the LAMP reaction in all samples was measured at OD 650 nm. The dotted black lines indicate the threshold value of turbidity (0.05) at which the LAMP reaction was considered positive. LAMP assays were based on target uvrD_2, while the qPCR targets for the multitarget assays included (**a**) IS*481* and (**b**) *ptx*S1.

To evaluate the colorimetric LAMP assay, we tested 600 DNA samples obtained from nasopharyngeal swabs collected from individuals who tested positive (n = 300) and negative (n = 300) for *B. pertussis* DNA according to multitarget qPCR (gold standard) at NHI-Peru. After parallel colorimetric LAMP and multitarget qPCR assays, 300/600 samples were confirmed to be positive for *B pertussis* DNA according to the diagnostic algorithm (online Supplementary Table S3), as evidenced by amplification of both IS*481* and *ptx*S1 with CT values ranging from 11.11 to 40.16 (Fig. 6a) and 18.24 to 39.27 (Fig. 6b), respectively. Of these samples, 299 were determined to be positive by the LAMP assay, suggesting a high sensitivity for a wide range of bacterial loads. LAMP products showed turbidities above 0.05 at 650 nm and were visually judged by a color change from brown to green after incubation for 60 min at 67 °C. In contrast, none of the negative samples in the LAMP assay changed in color or crossed the turbidity threshold (Fig. 6). Only one multitarget qPCR-positive specimen (with high CT values for IS*481* and *ptx*S1) was not detected by the LAMP assay, indicating a false negative (Fig. 6a and b). Overall, the sensitivity of the colorimetric LAMP assay was 99.7% (95% CI 98.2–100.0%), and the specificity was 100.0% (95% CI 98.8–100.0%) with a high degree of agreement between the two assays (Cohen’s kappa, 0.997; 95% CI 0.990–1.000; p < 0.0001). The PPV and NPV were 100.0% (95% CI 98.8–100.0%) and 99.7% (95% CI 98.2–100.0%), respectively.

### Colorimetric LAMP-positive samples, symptom onset and antibiotic treatment

**Figure 7:**
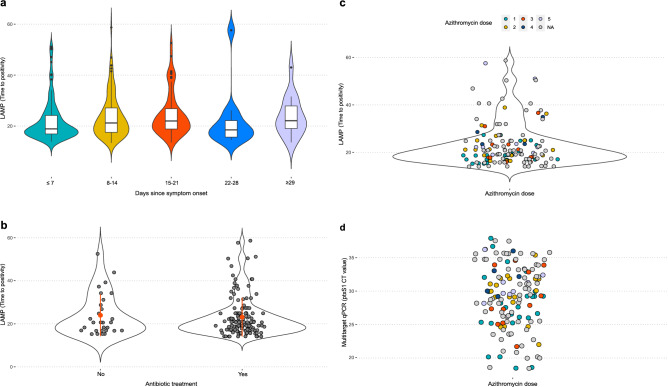
Colorimetric LAMP-positive samples over the time from symptom onset and antibiotic treatment from individuals with pertussis. (**a**) Violin plots show the time to positivity of the LAMP reaction over the days from symptom onset to sampling, (**b**) individuals with antibiotic treatment and (**c**) azithromycin doses administered to individuals with pertussis. (**d**) The dot plot shows the distribution of CT values for *ptx*S1 with qPCR according to the administered azithromycin dose. *NA* not available.

LAMP-positive samples had time to positivity (Tp) values ranging from 13.40 to 58.70 min that showed a significant positive correlation with the multitarget qPCR for the targets IS*481* (R = 0.73, p < 0.001) and *ptx*S1 (R = 0.76, p < 0.001) (online Supplementary Fig. S1). We also plotted LAMP Tp values against days since symptom onset, which were calculated as the time from the first symptom to sampling from individuals infected by *B. pertussis* (Fig. 7a). Violin plot analysis revealed that most isolates of *B. pertussis* (118/299) were detected by LAMP during the first week from symptom onset of individuals with pertussis (median = 5 days, range: 0–7 days) with a median Tp of 18.90 min (range: 13.90–51.10 min). In addition, the LAMP assay was able to identify *B. pertussis* in nasopharyngeal swabs obtained from individuals with pertussis with a broad period of symptom onset, including > 7 days and ≤ 14 days (100/299, median = 10 days); > 14 days and ≤ 21 days (54/299, median = 18 days); > 21 days and ≤ 28 days (12/299, median = 23.50 days); and ≥ 29 days (15/299, median = 34 days, range: 30–69 days) with a median Tp of 21.20 min (range: 13.40–58.70 min), 22.00 min (range: 13.60–52.60 min), 18.50 min (range: 14.60–57.60 min), and 22.10 min (range: 13.60–43.00 min), respectively (Fig. 7a). We also observed a high proportion of LAMP-positive specimens obtained from individuals who had received antibiotics (n = 138/299), including macrolides, penicillin, cephalosporins, and aminoglycosides (Fig. 7b). The most frequent antibiotic reported was azithromycin (n = 84/138), a first-line macrolide for the treatment of pertussis^39^. *B. pertussis* DNA was detected by LAMP in samples from patients who received up to 5 azithromycin doses with a median Tp of 20.00 min (range: 14.60–57.60 min) (Fig. 7c). A statistically significant difference (p = 0.010) was found between azithromycin ≤ 3 doses (54/68) and 4–5 doses (14/68) samples for CT values of *ptx*S1 multitarget qPCR. Most LAMP-positive samples with up to 3 azithromycin doses had a CT value for *ptx*S1 < 30 (37/54), while samples with high antibiotic doses (4 and 5) presented a CT value for *ptx*S1 > 28 (14/68), suggesting a low bacterial load (Fig. 7d).

## Discussion

Pertussis is a highly infectious respiratory disease caused by *B. pertussis*. Although pertussis vaccines are widely used in most countries, the resurgence of pertussis has become a major public health concern^40^. This resurgence has been observed in all age groups, mainly among infants younger than 3 months who are not yet vaccinated or partially immunized and who represent a group at higher risk for severe pertussis infection and death^41,42^. Remarkably, adults and adolescents, previously vaccinated as infants, are considered the most important sources of infection from pertussis, likely by waning immunity, which may result in asymptomatic transmission to susceptible populations^43^. Consequently, early and specific detection of *B. pertussis* is essential for timely treatment and preventing disease spread, especially during outbreaks. Nevertheless, most PCR-based methods use targets that are not *B. pertussis* specific. Recently, whole-genome sequencing-based pangenome analysis of multiple pathogens has provided a framework for identifying target genes to design highly specific and reliable diagnostic tests^44^. In this study, we developed and validated the feasibility of a colorimetric LAMP assay in a lyophilized format using a new and single target screened from the classical *Bordetella* pangenome for the specific and sensitive detection of *Bordetella pertussis*. Although classical *Bordetella* species such as *B. pertussis*, *B. parapertussis*, and *B. bronchiseptica* are highly similar at the genome sequence level^45^, we managed to identify highly specific targets that were present in ≥ 90% of a set of 861 *B. pertussis* isolates circulating worldwide but absent in the *B. bronchiseptica* and *B. parapertussis* genomes (Fig. 1). The optimized LAMP primer sets targeted the *uvrD_2* (uvrD_2), *group_18953* (MaoC), and *group_10409* (10409_hyp) genes, of which *urvD_2* was the unique gene that was not detected in any non-*B. pertussis* genomes. Although the *group_18953* and *group_10409* genes were identified in some *B. bronchiseptica* genomes but not in the *B. parapertussis* genome, they were selected to evaluate their performance on LAMP assays since they are present in 100% of analyzed *B. pertussis* genomes, and *B. bronchiseptica* isolates are rarely found in humans^46^. LAMP assays using primer sets uvrD_2, MaoC, and 10409_hyp efficiently amplified *B. pertussis* DNA and showed undetectable background amplification, with the best results obtained for the uvrD_2-based assay (Fig. 2). Several qPCR-based assays targeting IS*481*, the gene for outer membrane porin protein (*OMP*), the pertussis toxin (*ptx*) promoter region (*BPTP*), the pertactin gene (*PRN*), the putative thiolase gene (*BP283*), and the gene for porin protein (*POR*) have been described for the detection of *B. pertussis*^47–49^. IS*481* assays have shown higher sensitivity than non-IS*481* target assays but have also resulted in known cross-reactivity with other *Bordetella* species, whereas among the single-target qPCR assays, the POR assay has shown superior performance in specifically detecting *B. pertussis*, although clinical performance has been poorly evaluated since the number of PCR-positive clinical specimens tested has been too low (n = 20)^49^. In addition, the *POR* gene has highly homologous regions in the *B. parapertussis*, *B. bronchiseptica*, and *B. holmesii* genomes^49^, which makes it more complex for *B. pertussis*-specific primer design for isothermal amplification-based methods. Similarly, LAMP-based assays have previously been used to detect *B. pertussis,* typically targeting the IS*481* and *BPTP* genes^50^, with the same drawback reported in PCR methods. Our uvrD_2 LAMP assay with preheated template DNA showed higher sensitivity for detecting *B. pertussis* DNA in a sample with a low bacterial load (CT = 33.49) (Fig. 3). These results are in line with those of a previous study reported by Kamachi et al., in which BPTP-LAMP assays with heat-denatured template DNA were 100 times more sensitive than those with a nondenatured template^38^.

The uvrD_2 LAMP assay exhibited a LoD of 10 copies gEq × μl^−1^ with equal analytical sensitivity to *ptx*S1 with qPCR (10 copies gEq × μl^−1^) but slightly higher than the LoD of nonspecific-IS*481* with qPCR (1 copy gEq × μl^−1^) (Fig. 4). Furthermore, the uvrD_2 LAMP LoD was lower than that found in a study in which the LAMP assay targeting the BP485 region had a LoD of 1.3 pg/μl^50^, which is equal to approximately 250 genomic copies, assuming a *B. pertussis* Tohama genome size of 4.1 Mbp (2.4 genomic copies/10 fg DNA)^51^. Another study that evaluated a BPTP-LAMP assay coupled with a nanoparticle-based lateral biosensor reported an analytical sensitivity of 50 fg per reaction (~ 12 genomic copies)^40^, which is comparable to the LoD obtained using our colorimetric LAMP assay (10 copies gEq × μl^−1^ ~ 41 fg). However, genetic polymorphisms in *BPTP* have been identified among *B. pertussis* isolates circulating worldwide that differ from previously described *B. pertussis* isolates, which may lead to a further decrease in assay sensitivity and be a source of false negative results^52,53^. In addition, unlike the relative specificity for detecting *B. pertussis* due to pertussis toxin promoter that is also harbored by *B. parapertussis* and *B. bronchiseptica*^54^, the novel colorimetric LAMP assay based on uvrD_2 showed a high degree of specificity to *B. pertussis,* as evidenced by no cross-reactivity with *B. parapertussis*, *B. holmesii*, *B. bronchiseptica*, and other viruses and bacteria associated with respiratory infections (Fig. 5). These results were also confirmed by analytical in silico specificity of the uvrD_2 primer set, resulting in no match with any non-*B. pertussis* respiratory pathogens (online Supplementary Table S5), which makes the new assay highly specific for *B. pertussis* DNA detection in clinical samples.

The uvrD_2 LAMP assay proved to have high intra- and interassay precision across the dilutions tested, obtaining mean CV values that were lower than the recommended value of 15% (Tables 1 and 2)^55^. The mean CV value for the interassay precision of our LAMP assay was lower than the value reported for other LAMP assays based on the IS*481* sequence (4.14 vs. 7.38%, respectively), while intra-assay precision had a CV mean value comparable to that obtained using IS*481* LAMP (5.09 vs. 4.31%, respectively)^56^. Furthermore, our assay showed strong robustness through primer concentrations and temperatures, as demonstrated by another study that evaluated LAMP assays for detecting SARS-CoV-2^27^. Thus, our colorimetric LAMP assay can provide substantial sensitivity and robustness with high precision for *B. pertussis* detection.

The colorimetric uvrD_2 LAMP assay showed a sensitivity of 100.0% (95% CI 97.4–100.0%) and specificity of 98.6% (95% CI 94.9–99.8%), with almost perfect agreement (Cohen’s kappa, 0.997; 95% CI 0.990–1.000; p < 0.0001), compared to the reference test, suggesting that our LAMP assay is highly comparable to expensive multitarget qPCR to specifically and sensitively detect *B. pertussis* DNA in nasopharyngeal clinical specimens (Fig. 6)^57^. Likewise, the uvrD_2 LAMP assay resulted in higher diagnostic sensitivity and specificity than those reported in previous LAMP assays targeting IS*481* [87.5% (95% CI 78.2−93.8% and 92.9% (95% CI 85.2−97.3%)] and the *ptx* promoter region [76.2% (95% CI 65.4−85.0%) and 94.1% (95% CI 86.8−98.0%)]^29^. In our study, we obtained only one false negative (Fig. 6), likely due to the high specificity of the uvrD_2 LAMP primers for detecting *B. pertussis* and misdiagnosis of *B. bronchiseptica* as *B. pertussis* by multitarget qPCR^58^.

Unlike other diagnostic methods based on culture and PCR, our uvrD_2 LAMP assay was able to detect *B. pertussis* DNA in clinical specimens from individuals with a wide range of bacterial loads and symptom onset times, providing detection at both early and later stages of disease (Figs. 6 and 7a)^59^. Since pertussis is highly contagious, treatment with antibiotics is recommended to avoid the severity and transmission of the disease. However, antibiotic therapy may decrease the likelihood of a positive result^59^. Our LAMP assay managed to amplify *B. pertussis* DNA in samples from individuals who had received different antibiotics, mainly up to 5 azithromycin doses (Fig. 7b–d). These results are in line with those obtained in a study that assessed the persistence of *B. pertussis* DNA by means of IS*481* real-time PCR using nasopharyngeal aspirates from 22 antibiotic-treated children, obtaining an 83% positivity rate after 14 days of erythromycin treatment^60^. Conversely, in a prospective trial, Pichichero et al. found that all 29 patients diagnosed with pertussis by culture were PCR negative after 3 days of azithromycin treatment^61^. Differences between these results likely depend on the clinical picture, used antibiotic and other factors, such as resistance to antibiotics^23^. Overall, the *B. pertussis* DNA load decreases gradually during antibiotic therapy^60^, as was shown in this study, in which LAMP-positive samples obtained CT values for *ptx*S1 > 28 at high azithromycin doses (Fig. 7d).

In conclusion, the novel colorimetric uvrD_2 LAMP assay informed by the pangenome is highly sensitive and specific for detecting *B. pertussis* DNA in nasopharyngeal swabs, with exceptionally similar diagnostic performance to that of complex and high-cost multitarget qPCR, but it is faster, simpler, and inexpensive. Our results also revealed that the assay is able to identify *B. pertussis*-infected individuals at both early and later stages of disease, many of whom have received antibiotic therapy. This makes the new colorimetric LAMP assay very helpful for the reliable and timely diagnosis of pertussis infection, as well as for strengthening epidemiologic surveillance in primary health care to control and prevent the disease.

## Supplementary Information

Supplementary Information.

Supplementary Table S1.

## Data Availability

Study information is included in the article and Supplementary Material. Further inquiries can be made to the corresponding author. Sequencing reads generated for this study can be found in NCBI Sequence Read Archive under BioProject accession number: PRJNA883367 (https://www.ncbi.nlm.nih.gov/sra).
