# Supplementary material for: A pangenome approach-based loop-mediated isothermal amplification assay for the specific and early detection of Bordetella pertussis
Source: Sci Rep. 2023 Mar 16;13:4356. doi: 10.1038/s41598-023-29773-9 (PMC10018623; doi:10.1038/s41598-023-29773-9)
Supplement: Supplementary file 1 — Supplementary Information. [file 41598_2023_29773_MOESM1_ESM.docx]

A pangenome approach-based loop-mediated isothermal amplification assay for the specific and early detection of *Bordetella pertussis*

Eduardo Juscamayta-López^1,2,*^, Faviola Valdivia^1^, María Pía Soto^1^, Brenda Nureña^1^, Helen Horna^1^

^1^Laboratorio de Infecciones Respiratorias Agudas, Centro Nacional de Salud Pública, Instituto Nacional de Salud, Lima, Peru

^2^Facultad de Salud Pública y Administración (GA, AGL), Universidad Peruana Cayetano Heredia, Lima, Peru

*** Correspondence:**Eduardo Juscamayta-López
jjuscamamayta@ins.gob.pe, ejuscamaytal@gmail.com


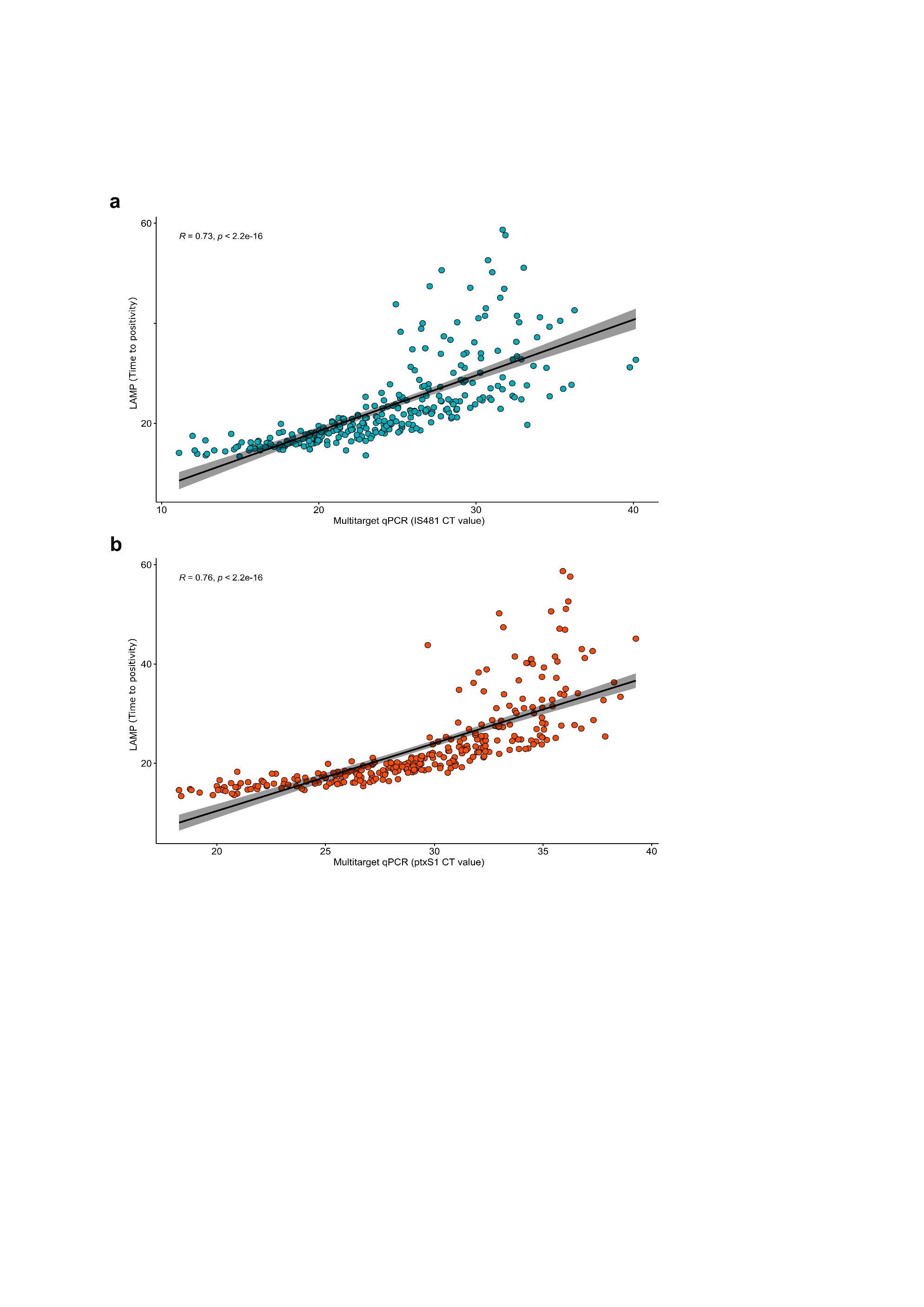


**Supplementary Fig. S1.** Correlation between the time to positive LAMP assay and CT values of the multitarget qPCR assay in nasopharyngeal swab samples. The values on the y-axis are the time to positivity (in minutes), which was defined as the time at which the LAMP reaction color changed from brown to green and the turbidity increased above 0.05 within 60 min. LAMP assays were based on target uvrD_2, while the qPCR targets for the multitarget assays included (A) IS*481* and (B) *ptx*S1. Nasopharyngeal swab specimens (n=299) were positive for *Bordetella pertussis* DNA using both LAMP and multitarget qPCR assays.


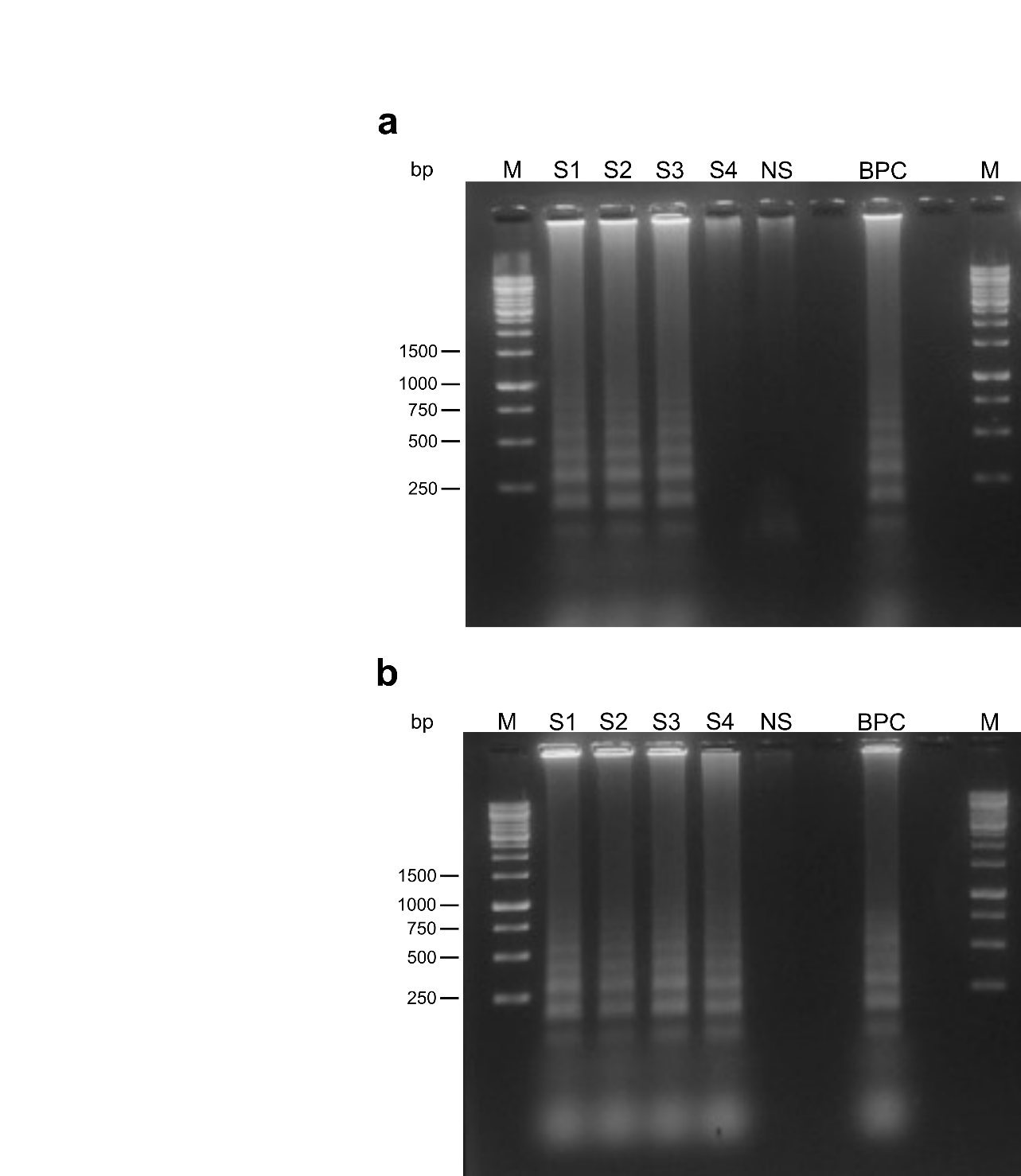


**Supplementary Fig. S2.** Original agarose gels after electrophoresis of uvrD_2-LAMP products using (a) nonpreheated and (b) presheated template DNA from a clinical sample panel with a bacterial load gradient showed in Fig. 1. Preheated DNA consisted of denaturing template DNA at 96 °C for 5 min prior to loading of the sample for the LAMP reaction. Reactions were considered positive for LAMP products if they had both a color change from brown to green and a ladder-like banding pattern on agarose gel after electrophoresis. S1-S4, clinical samples positive for *B. pertussis* DNA with different cycle threshold (CT) values obtained by multitarget qPCR (CT_S1_=11.93, CT_S2_=16.39, CT_S3_=25.80, and CT_S4_=33.49); NS, clinical samples negative for *B. pertussis* DNA; BPC, *B. pertussis* positive control; M, molecular marker; bp, base pair.

**Supplementary Table S2.** Sequences in each LAMP primer set designed for detecting *Bordetella pertussis*.

| **Primer** | **Target** | **Name** | **Sequence (5'–3')** | **bp** |
| --- | --- | --- | --- | --- |
| F3 | uvrD_2 | Hypothetical protein | CACTTTCACCACCAGTCTGG | 20 |
| B3 |  |  | CGACATAGCAGAGCAGCAG | 19 |
| FIP |  |  | TGCCGTTGCCAATAGGATCCATATGAACAAAAGGCCGCCA | 40 |
| BIP |  |  | CCGCGCCAAGGATCCGAATTCGGTCTTATGGACGATCAGG | 40 |
| LF |  |  | TGCAAGTCGAAGGCGGTG | 18 |
| LB |  |  | TCTGGTCGGTGCGAGTC | 17 |
| F3 | group_18953 | MaoC family protein | CACTCACGGGGAACTGGA | 18 |
| B3 |  |  | TTTCTCATGACCTCGCAGAC | 20 |
| FIP |  |  | ACGTCAACGACCCTTGCACCAATGTGCAATACGCGTCCAA | 40 |
| BIP |  |  | GGCCTTGGCATTCAGGCGAATAGATGGTGTCGCCGATGG | 39 |
| LF |  |  | CGCTTGCCGAAACGTGTC | 18 |
| LB |  |  | CATGCGGTTTCTGGCTCCG | 19 |
| F3 | group_10409 | Hypothetical protein | GCCTGGTACCGAAAAGAGG | 19 |
| B3 |  |  | GCGAATGCCATACGTCCTT | 19 |
| FIP |  |  | CTTTCCTCGGCACGCTTCTCCTATCTCGAACGACGCCCAT | 40 |
| BIP |  |  | GGACACGTCGTCATCAAGGCAGGCGTCGACTTTGTATCTGT | 41 |
| LF |  |  | TCCTCGAAGGTATCGGGGAA | 20 |
| LB |  |  | ACATCGACCCAGATACATTCCC | 22 |

**Supplementary Table S3.** Diagnostic algorithm for the interpretations of the multitarget real-time PCR results.

| Interpretation | Assay results^†^ | | | |
| --- | --- | --- | --- | --- |
|  | Multiplex | | | Singleplex |
|  | IS*481* | pIS*1001* | hIS*1001* | *ptxS1* |
| *B. pertussis* | Positive  (*Ct*<35) | Negative | Negative | Positive  (*Ct≤40*) |
| *B. pertussis* | Positive  (*Ct*≥35) | Negative | Negative | Positive  (*Ct≤40*) |
| *B. holmesii* | Positive  (*Ct*<35) | Negative | Positive (*Ct≤35*) | Negative |
| *B*. *parapertussis* | Negative | Positive  (*Ct≤35*) | Negative | Positive  (*Ct≤36*) |
| Indeterminate^††^ | Positive  (*Ct*≥35) | Negative | Negative | Negative |

^†^Algorithm based on the multitarget qPCR assay described by Tatti et al. ^21^

^††^Requires confirmation by other methods, such as culture, serology, or epidemiologic linkage.

**Supplementary Table S4.** Primers and probes used in multitarget real-time PCR (gold standard) for detecting *Bordetella pertussis*.

| **Target** | **Primer/probe** | **5' fluorophore** | **Sequence** | **3' quencher** | **Concentration (µM)** |
| --- | --- | --- | --- | --- | --- |
|  |  |  |  |  |  |
| IS*481* | 852U18_F |  | CAAGGCCGAACGCTTCAT |  | 0.1 |
|  | 894L24_R |  | GAGTTCTGGTAGGTGTGAGCGTAA |  | 0.1 |
|  | 871U22P_P | FAM | CAGTCGGCCTTGCGTGAGTGGG | BHQ1 | 0.3 |
| hIS*1001* | BHIS41U20_F |  | GGCGACAGCGAGACAGAATC |  | 0.1 |
|  | BHIS91L17_R |  | GCCGCCTTGGCTCACTT |  | 0.1 |
|  | BHIS62U28P_P | Cy5 | CGTGCAGATAGGCTTTTAGCTTGAGCGC | BHQ3 | 0.1 |
| pIS*1001* | 135U17_F |  | TCGAACGCGTGGAATGG |  | 0.3 |
|  | 199L20_R |  | GGCCGTTGGCTTCAAATAGA |  | 0.3 |
|  | 157U21P_P | HEX | AGACCCAGGGCGCACGCTGTC | BHQ1 | 0.1 |
| *ptx*S1 | 402U16_F |  | CGCCAGCTCGTACTTC |  | 0.7 |
|  | 442L15_R |  | GATACGGCCGGCATT |  | 0.7 |
|  | 419U22P_P | Texas Red | AATACGTCGACACTTATGGCGA | BHQ2 | 0.3 |
| *rnaseP* | rnaseP_F |  | CCAAGTGTGAGGGCTGAAAAG |  | 0.3 |
|  | rnaseP_R |  | TGTTGTGGCTGATGAACTATAAAAGG |  | 0.3 |
|  | rnaseP_P | Cy5.5 | CCCCAGTCTCTGTCAGCACTCCCTTC | BHQ3 | 0.1 |

**Supplementary Table S5.** Pathogens associated with respiratory infections and Bordetella species closely related to *Bordetella pertussis* used in the analytical in silico specificity evaluation of the final LAMP primer set.

| **Respiratory pathogen** | **GenBank ID** |
| --- | --- |
| *Bordetella bronchiseptica* | NZ_CP020819 |
| *Bordetella holmesii* | NZ_CP007158 |
| *Bordetella parapertussis* | NZ_CP020654 |
| *Haemophilus influenzae* | NC_017452 |
| *Haemophilus parainfluenzae* | NC_015964 |
| *Streptococcus pneumoniae* | NC_003028 |
| *Streptococcus pyogenes serogroup A* | NC_011375 |
| *Streptococcus agalactiae* | NZ_CP007632 |
| *Neisseria meningitidis serogroup B* | NC_017513 |
| *Corynebacterium diphtheriae* | NC_016785 |
| *Legionella pneumophila* | NZ_CP015344 |
| *Chlamydophila pneumoniae* | NC_005043 |
| *Mycoplasma pneumoniae* | NC_000912 |
| *Escherichia coli* | NZ_CP080120 |
| *Aggregatibacter aphrophilus* | NZ_CP009230 |
| *Klebsiella pneumoniae* | NZ_CP040363 |
| *Pseudomonas aeruginosa* | NZ_CP041354 |
| *Staphylococcus aureus* | NZ_CP080548 |
| *Mycobacterium tuberculosis* | NZ_CP072764 |
| Respiratory syncytial virus | NC_001803 |
| Influenza A virus | NC_007373 |
| Parainfluenza virus 1 | NC_003461 |
| Adenovirus | NC_012959 |
| SARS coronavirus ZMY 1 | AY351680 |
| SARS-CoV-2 | NC_045512 |
| Human coronavirus OC43 | NC_006213 |
| Human metapneumovirus (hMPV) | NC_039199 |
| Enterovirus | NC_013695 |
| Rhinovirus | NC_038312 |
